# Nile Red Quantifier: a novel and quantitative tool to study lipid accumulation in patient-derived circulating monocytes using confocal microscopy[S]

**DOI:** 10.1194/jlr.D073197

**Published:** 2017-09-28

**Authors:** Johan G. Schnitzler, Sophie J. Bernelot Moens, Feiko Tiessens, Guido J. Bakker, Geesje M. Dallinga-Thie, Albert K. Groen, Max Nieuwdorp, Erik S. G. Stroes, Jeffrey Kroon

**Affiliations:** Departments of Vascular Medicine* University of Amsterdam, Amsterdam, The Netherlands; Experimental Vascular Medicine,† Academic Medical Center, University of Amsterdam, Amsterdam, The Netherlands; Department of Pediatrics,§ Laboratory of Metabolic Diseases, University of Groningen, University Medical Center Groningen (UMCG), Groningen, The Netherlands; Wallenberg Laboratory,** University of Gothenberg, Gothenberg, Sweden; Department of Internal Medicine, Diabetes Center,†† Vrije Universiteit (VU) University Medical Center, Amsterdam, The Netherlands; Institute for Cardiovascular Research,§§ Vrije Universiteit (VU) University Medical Center, Amsterdam, The Netherlands

**Keywords:** lipid droplets, methods, cholesterol and trafficking, fluorescence and confocal imaging

## Abstract

The inflammatory profile of circulating monocytes is an important biomarker for atherosclerotic plaque vulnerability. Recent research revealed that peripheral lipid uptake by monocytes alters their phenotype toward an inflammatory state and this coincides with an increased lipid droplet (LD) content. Determination of lipid content of circulating monocytes is, however, not very well established. Based on Nile Red (NR) neutral LD imaging, using confocal microscopy and computational analysis, we developed NR Quantifier (NRQ), a novel quantification method to assess LD content in monocytes. Circulating monocytes were isolated from blood and used for the NR staining procedure. In monocytes stained with NR, we clearly distinguished, based on 3D imaging, phospholipids and exclusively intracellular neutral lipids. Next, we developed and validated NRQ, a semi-automated quantification program that detects alterations in lipid accumulation. NRQ was able to detect LD alterations after ex vivo exposure of isolated monocytes to freshly isolated LDL in a time- and dose-dependent fashion. Finally, we validated NRQ in patients with familial hypercholesterolemia and obese subjects in pre- and postprandial state. In conclusion, NRQ is a suitable tool to detect even small differences in neutral LD content in circulating monocytes using NR staining.

Atherosclerosis is characterized by the recruitment of inflammatory cells into the intima, where excessive uptake of modified LDL particles, such as oxidized LDL, by monocytes and macrophages leads to foam cell formation (1), promoting the initiation of atherosclerotic lesions. Subsequent endothelial activation leads to continuous recruitment of circulating monocytes into the subintimal area, further increasing inflammatory activity of these lesions, leading to plaque progression and, eventually, plaque destabilization (2–4). Recently, it has been suggested that migration of circulating monocytes is not merely a reflection of local endothelial activation near atherogenic areas, but circulating monocytes may also display pro-adhesive properties in patients at increased cardiovascular risk (5). The factors leading to this systemic activation of circulating monocytes remain to be established. In dyslipidemic states, lipid accumulation is thought to contribute to the pro-adhesiveness of the circulating cells. In the postprandial state, lipids were found to be taken up by circulating monocytes, resulting in increased expression of the integrin CD11c, binding partner of vascular adhesion molecule-1 (VCAM-1) (6), as well as increased monocyte-derived production of tumor necrosis factor α and interleukin 1β (7). In this context, circulating monocytes may serve as a biomarker for atherosclerotic plaque vulnerability (1, 8, 9), whereas lipid accumulation in these cells may be an important driver for this systemic pro-inflammatory phenotype (6, 7, 10).

In cells, lipids are usually stored in lipid droplets (LDs) (11, 12). LDs have been identified as organelles that are actively involved in many essential cellular functions, such as cellular homeostasis (membrane biogenesis and lipoprotein synthesis), cell survival in a nutrient-poor milieu, protein storage, and protection against lipotoxicity by scavenging free fatty acids and cholesterol (12). LDs consist of a surrounding phospholipid layer, with similar composition to that of the ER membrane, and it encloses a neutral lipid core of cholesterol esters (CEs) and triglycerides (TGs) in various ratios (13). Lipid uptake must be tightly controlled in order to maintain cellular homeostasis by preventing lipid overload and subsequent cellular activation (14).

A number of techniques have been developed to detect lipids in cytoplasmic LDs (15, 16). One of the best known lipid dyes used to assess cytoplasmic lipid content in cells is Oil Red O (ORO), although it has become evident that ORO has multiple drawbacks (17, 18). Because ORO is very sensitive to preparation conditions, working solutions must be freshly prepared, which is labor intensive (19), but more importantly, quantification of lipid particles is arbitrary (15). Another frequently used lipid dye is filipin, but it is known to photobleach rapidly (20), whereas the fluorophore, BODIPY, can bind nonspecifically to other intracellular structures (16).

Here, we set out to develop a fast and quantitative method to quantify neutral LDs in circulating monocytes stained with the lysochrome, Nile Red (NR), first described in peritoneal macrophages by Greenspan, Mayer, and Fowler (21) in 1985. Greenspan and others considered NR as an environment-sensitive fluorescent dye used to detect intracellular lipid particles with high sensitivity (21, 22) in multiple cell types (11) by high-resolution confocal laser scanning microscopy. For quantification of the cytoplasmic neutral LDs, we developed Nile Red Quantifier (NRQ), a MATLAB-based freely available program for quick and easy assessment of lipid uptake.

## MATERIALS AND METHODS

### Monocyte isolation

The study was approved by the institutional review board of the Academic Medical Center (AMC) and conducted according to the Declaration of Helsinki. Written informed consent was obtained from all participants. Venous blood was drawn in EDTA-coated Vacutainer® tubes (BD, Plymouth, UK) to prevent coagulation. Peripheral blood mononuclear cells were obtained from whole blood samples through density centrifugation using Lymphoprep (d = 1.077), as described in detail elsewhere (23). In short, blood was diluted in a 1:1 ratio with PBS/2 mM EDTA and subsequently added to a layer of Ficoll-Paque PLUS (Axis-Shield). Next, cells were centrifuged for 20 min at 600 *g* at room temperature with slow acceleration and no brake. The peripheral blood mononuclear cell fraction was collected and washed twice with PBS/2 mM EDTA. Next, cells were counted using a CASY counter (CASY TT; Roche Innovatis, Bielefeld, Germany). Subsequently, monocytes were isolated using human CD14 magnetic beads and MACS® cell separation columns according to the manufacturer’s protocol (Miltenyi, Bergisch Gladbach, Germany). In short, cells were resuspended in MACS buffer [0.5% bovine serum albumin (Sigma-Aldrich, St. Louis, MO) in PBS/2 mM EDTA]. Next, CD14 MicroBeads (Miltenyi Biotec, Leiden, The Netherlands) were added and incubated for 30 min at 4°C. After incubation, the cells were washed with MACS buffer and resuspended in MACS buffer for CD14 bead isolation using MACS separation columns (Miltenyi Biotec). After isolation, cells were counted by means of a CASY counter. Monocytes were kept in Iscove’s modified Dulbecco’s medium (product number 12440-53; Thermo Fisher Scientific, Waltham, MA), without any additives and containing no proteins, lipids, or growth factors, for further immunohistochemistry and immunofluorescent analysis. All experiments were exclusively performed with freshly isolated circulating monocytes.

### LD and neutral lipid extraction

LD extraction from peripheral monocytes was performed on 10 × 10^6^ CD14+ monocytes according to the manufacturer’s instructions (#MET-5011; Cell Biolabs Inc., San Diego, CA). Next, density gradient ultracentrifugation in a SW41 rotor (Beckman, Uithoorn, The Netherlands) was performed for 3 h at 20,000 *g* at 4°C. The top layer, containing the floating LDs, was collected and used for neutral lipid extraction. Lipids were isolated, as previously described, by performing a butanol-methanol extraction (24). Neutral lipids were separated from polar lipids by dissolving the nitrogen-dried lipid extract in heptane:methanol (98:2). After vortexing, 300 μl methanol:water + 1% NH_3_ (of 23% NH_3_ stock) were added and the upper phase containing the neutral lipids was collected and dried at 35°C under a continuous flow of nitrogen gas.

### HPLC analysis

The neutral lipid extractions were analyzed using a LC-4000 series UHPLC system (Jasco, Tokyo, Japan). A 5 ul volume of sample was injected with a Jasco AS-4250 UHPLC autosampler (Jasco) on a Spherosorb 5.0 μM silica column (4.6 × 100 mm) (Waters, Dublin, Ireland) under a constant temperature of 45°C. A linear gradient was applied on the mobile phase by mixing phase A in 9 min from 0% to 50% with phase B at a continuous flow of 1.6 ml/min. Phase A consisted of a heptane/ethyl acetate (98.8/0.2%), whereas phase B consisted of an acetone/ethylacetate (2:1) mixture (Merck Chemicals, Amsterdam, The Netherlands). Finally, the relative peak areas of the cholesterol and TGs were calculated using Chromnav v 2.0 software (Jasco).

### LDL isolation by ultracentrifugation

Lipoprotein fractions were isolated from plasma of healthy normolipidemic volunteers (both male and female from different ages) in EDTA-containing 10 ml Vacutainer® tubes. Human LDL (d = 1.019–1.063 g/l) was isolated from plasma by density gradient ultracentrifugation, as described previously (25). Briefly, plasma was obtained after centrifugation of blood at 2,486 *g* at 4°C for 15 min. Next, 1,155 mg of potassium bromide and 75 mg of sucrose were dissolved in 3 ml of plasma to a final density of 1.25 g/ml. Then, 3 ml of high density plasma was pipetted into a polystyrene SW41 tube (Beckman). The gradient was built by carefully consecutively pipetting 2.0 ml KBr solution with d = 1.225, 4 ml KBr (d = 1.100 g/ml), and 3 ml KBr (d = 1.006 g/ml) on top of the plasma. Ultracentrifugation was run at 144,200 *g*, slow acceleration and slow brake, for 19 h at 10°C in an Optima XPN-100 ultracentrifuge system (Beckman, Fullerton, CA). After centrifugation, LDL fractions were harvested by tube slicing and cholesterol, TGs, and apoB concentrations were measured using commercially available enzymatic assays (Diasys, Holzheim, Germany) on a Selectra system (Sopachem, Ochten, The Netherlands). Prior to the experiments, the fractions were dialyzed for 24 h in PBS, which was changed at least three times. Next, the isolated and dialyzed LDL samples were filter sterilized using 0.45 μm Minisart® syringe filters (Sartorius, Göttinger, Germany). Incubation experiments with LDL were normalized to apoB concentrations. For the dose-response and time incubation studies, monocytes were incubated on fibronectin-coated (30 μg/ml) glass microscope slides with either an increasing concentration of freshly isolated LDL for 1 h at 37°C, 5% CO_2_ or a fixed concentration (50 μg/ml) of LDL for an increasing period of time at 37°C, 5% CO_2_. Only freshly isolated LDL was used to minimize LDL oxidation due to storage conditions. In order to maintain similar volumes, Iscove’s modified Dulbecco’s medium without any additives and without proteins, lipids, or growth factors was added to a final volume of 200 μl. After incubation, monocytes were fixed with 4% formaldehyde.

### NR lipid content staining of CD14+ monocytes

The NR (9-diethylamino-5H-benzo[α]phenoxazine-5-one) staining solution (1 mM) was prepared by dissolving 318 μg/ml NR in DMSO and filtering through a 0.22 μm Minisart® syringe filter (Sartorius) in order to reduce fluorescent background. Aliquots were stored at −20°C for at least 6 months. The working solution was prepared freshly by dissolving NR stock solution in an isosmotic and isotonic solution (BD FACS buffer) to a final concentration of 10 μM that was protected from light.

Isolated monocytes were plated on fibronectin-coated glass microscope slides after drawing a circle of approximately 1.5 cm with a DAKO hydrophobic pencil (Dako, Heverlee, Belgium). Next, 200 μl of monocyte suspension (concentration of 0.5 × 10^6^/ml) was placed on the glass slide and incubated for 1 h at 37°C, 5% CO_2_, after which the adhered cells were fixed with a final concentration of 4% formaldehyde. Next, the cells were washed with PBS and stored at 4°C. Monocytes were then permeabilized for 5 min with 0.1% Triton X-100 and incubated with the lipid dye, NR (1 μg/ml, N3013-100MG; Sigma-Aldrich, Zwijndrecht, The Netherlands) for 15 min. Cells were washed in PBS (pH 7.4) and mounted using fluorescent mounting medium (Dako).

### Image acquisition

Imaging was performed on a Leica TCS SP8 confocal laser scanning DMI6000 inverted microscope (Leica Microsystems, Wetzlar, Germany) using a 63×/1.40 oil CS2 objective coupled to a Hamamatsu camera. Phospholipids were excited at 590 nm (600–700 nm) and neutral lipids at 488 nm (500–580 nm). In addition, line averaging was used during image acquisition to minimize background noise. Quantification of LDs was performed by assessing the total number of monocytes with LDs per field of view (FOV), as well as the number of LDs per positive monocyte in 5–10 FOVs. In order to visualize all LDs in the cell, resolution was significantly enhanced through a digital magnification of 1.5–2, z-stack images of 0.75 μm per stack (1,024 × 1,024 pixels × 8 bit), and subsequently merged into a maximum intensity projection for quantification. A minimal number of eight images per sample were taken. For intracellular LD assessment, an extensive z-stack was made of NR-stained monocytes. Subsequently, for 3D imaging, the software program, Imaris® (version 7.7.0; Belfast, UK), was used for rendering these extensive z-stack images into a 3D model.

### Quantification of LDs using MATLAB™ AMC NRQ

The cell and lipid counter, named NRQ, is a custom-made stand-alone program with in-house-created code in MATLAB^TM^ 2015b (The MathWorks Inc., Natick, MA). NRQ is based on semi-automated segmentation, where the user has to point out and click on the cells in the image, leaving a mark for recognition. With this program, the total number of lipids can be counted automatically, as well as the total number of cells and the number of lipid-positive cells per FOV. Obtained microscopic images (RGB color, TIFF format) with a resolution of 1,024 × 1,024 pixels are first converted into grayscale. This step ensures that luminance is retained, whereas it eliminates hue and saturation. This grayscale image is morphologically opened (26) using a disk structure with a user-defined size and, subsequently, the image is contrast enhanced. The 2D cell segmentation is performed and enables monocyte detection, as well as removal of monocytes touching the edges, because lipid count is incomplete in these cells. This binary mask, dilated with a user-defined size, is visualized on the original image to check to determine whether LDs are within these masks. LDs are segmented by creating a binary lipid mask, as described earlier. The hue, saturation, and value (HSV) segmentation method is used to identify boundaries of objects, making it suitable for segmentation. This enables the detection of LDs, for which value (color range) is modifiable to user preference or dye. Next, the background correction is applied by subtracting the inverse monocyte cell mask from the LD mask to avoid possible lipids located outside cell regions. Although LDs in isolated monocytes are spherically shaped, in general, the space between LDs can be small and segmentation can lead to overlapping lipids. To distinguish individual LDs from connected LDs in the mask, shape measurements are computed. Subsequently, the original image with the transparent monocyte cell-mask is displayed. A new binary monocyte cell mask, with a user-defined octagonal size, is created using these marks. Detailed descriptions regarding the MATLAB code can be found in the supplemental data.

### Validation of NR lipid content assessment using ORO

The ORO staining solution was made by dissolving 50 mg of ORO in 10 ml isopropanol and incubating overnight at 56°C. Next, the stock solution was filtered through standard Whatman® filter paper (Sigma-Aldrich) and was kept at room temperature. The working solution was prepared by mixing six parts of ORO stock and four parts of ultrapure water and subsequently filtering through standard Whatman® filter paper to remove precipitates. After monocyte isolation, cells were fixed with a final concentration of 4% formaldehyde and subsequently permeabilized for 5 min using 0.1% Triton X-100. Next, coverslips were incubated in 60% isopropanol for 5 min and subsequently incubated for 15 min with ORO. Cells were destained with 60% isopropanol for 15 s, washed once with water, and mounted. Imaging was performed on a Leica DM-RA wide-field microscope using a PLAN APO 40×/1.40 oil PH3 objective and a charge-coupled device camera (Leica Microsystems) or a Leica TCS SP8 confocal laser scanning DMI6000 inverted microscope for fluorescent ORO assessment (excited at 568 nm).

### Culturing THP-1 cells

The human THP-1 monocytic cell line (27) (ATCC, Manassas, VA) was used as an in vitro model for investigating LD-formation and inflammatory response upon LDL exposure. THP-1 cells were cultured in RPMI 1640 GlutaMAX-medium (Thermo Fisher Scientific) supplemented with 10% FBS (Bodinco, Alkmaar, The Netherlands), penicillin (100 U/ml; Thermo Fisher Scientific), and streptomycin (100 mg/ml; Thermo Fisher Scientific) at 37°C at 5% CO_2_. At least 24 h prior to the experiments, THP-1 cells (passage 2–8) were incubated in RPMI-medium containing penicillin/streptomycin and 1% lipoprotein-depleted FBS (Bodinco). Cells were adhered to a microscope slide using a Cytospin cytocentrifuge (Thermo Fisher Scientific) for 2 min at 34 *g*. After fixation, cells were incubated with NR for subsequent neutral LD analysis.

### Clinical validation

Previous studies reported the presence of lipid-laden monocytes in a setting of postprandial hyperlipidemia (6), genetically elevated levels of remnant cholesterol (28), and familial hypercholesterolemia (FH) (29). To assess the applicability of the NRQ in disease settings, we included pilot analyses of: *a*) three heterozygous FH patients (30) currently not receiving therapy due to statin-associated muscle symptoms (31) and compared them to age and gender matched healthy controls and *b*) three obese subjects who underwent an oral fat load. Briefly, participants were admitted to the hospital and blood was drawn in a fasting state for neutral LD assessment. Subsequently, cream consisting of 40% fat (v/v) was administered in a dose of 50 g fat per square meter of body surface and blood was drawn after 4 h for postprandial neutral LD assessment. Patient characteristics are listed in **Table 1**. In plasma of all subjects, a lipid profile was measured using standard laboratory procedures. Monocytes were isolated for neutral LD content assessment, as described above.

**TABLE 1.** Baseline characteristics of the clinical validation of NRQ

	Healthy Control (n = 3)	FH (n = 3)	*P*	Pre-meal	Post-meal	*P* (Pre-meal vs. Post-meal)
Age, years	60 ± 10	61 ± 12	0.908	56 ± 12	n/a	n/a
Gender, n, male (%)	3 (100)	3 (100)	n/a	3 (100)	n/a	n/a
BMI	n/a	n/a	n/a	38.3 ± 5.27	n/a	n/a
Total cholesterol, mmol/l	5.2 ± 0.9	8.8 ± 0.6	0.005	4.75 ± 0.11	4.97 ± 0.07	0.056
LDL-C, mmol/l	3.4 ± 0.6	6.9 ± 0.7	0.002	n/a	n/a	n/a
HDL-C, mmol/l	1.4 ± 0.3	1.0 ± 0.2	0.130	n/a	n/a	n/a
TGs, mmol/l	1.45 ± 0.3	1.78 ± 0.1	0.126	2.13 ± 0.29	3.27 ± 0.85	0.103

Values are n (%) or mean ± SD. HDL-C, HDL cholesterol; LDL-C, LDL cholesterol; n/a, not applicable.

### Statistical analysis

Data are presented as the mean ± SEM for continuous variables and as number (percentage) for categorical variables, unless stated otherwise. Correlations were assessed using univariate linear regression. Time- and dose-dependency experiments were assessed with a one-way ANOVA Dunnett’s method for multiple comparisons. The agreement between experiments and analyses were assessed using intraclass correlation coefficients (ICCs; r). The SD of the paired differences and the coefficient of variation were calculated. The coefficient of variation was calculated by dividing the SD of the paired differences by the mean value of the sample. All other experiments were analyzed using the Student’s *t*-test. All statistics were assessed using GraphPad Prism (v6.0 h; La Jolla, CA) and statistical significance was reported for *P* < 0.05.

## RESULTS

### NR imaging in peripheral human CD14+ monocytes

**Fig. 1.**
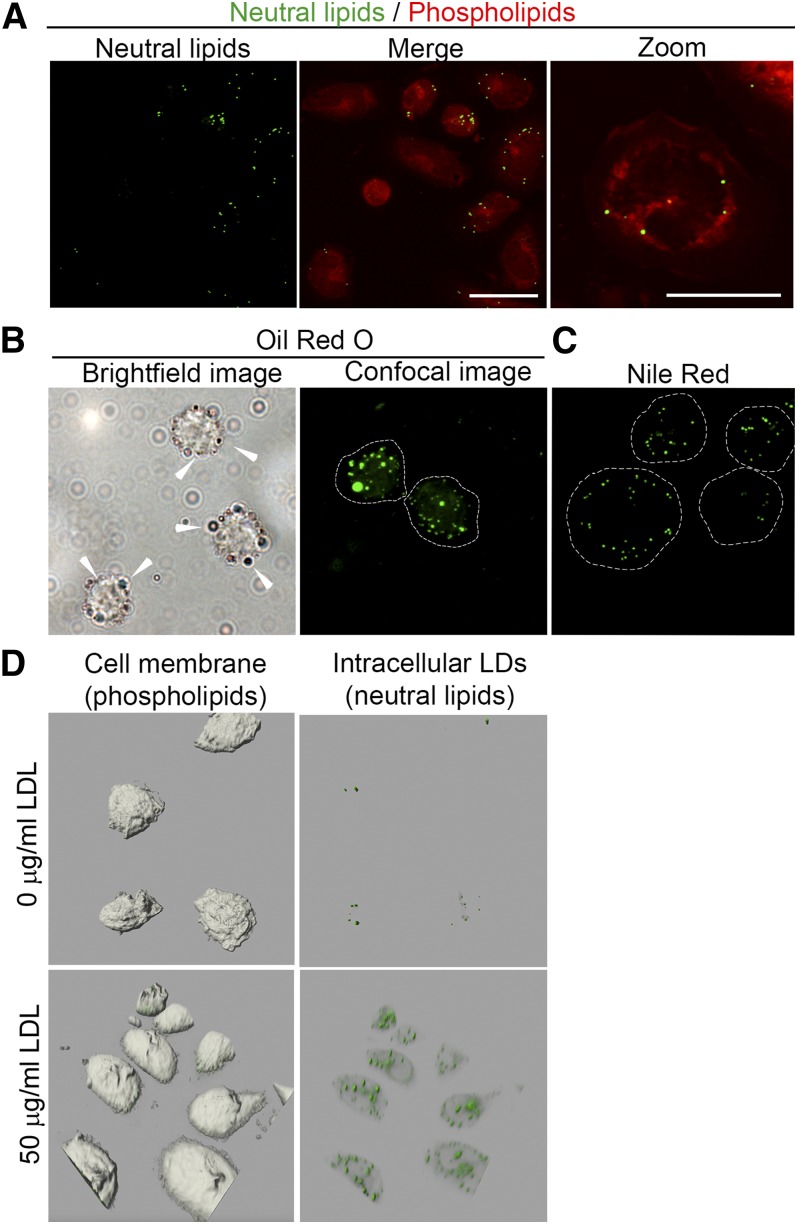
LD detection by NR in human peripheral monocytes. A: NR-stained monocytes. Neutral lipids (green spheroids; left image) can be discriminated from phospholipids (red; middle image and right image; scale bars, 20 and 10 μm, respectively). B: Representative images of ORO-stained monocytes incubated with 50 μg/ml LDL for 1 h. The left image shows LDs by bright-field microscopy; LDs are indicated with white arrows. The right image was obtained by confocal microscopy. LDs are shown in green. C: NR-stained monocytes treated with 50 μg/ml LDL for 1 h. LDs are shown in green. Scale bar, 10 μm. D: The 3D images of monocytes (gray bodies shown on left images) untreated (upper images) and treated (lower images) for 1 h with 50 μg/ml LDL. LDs are shown in green.

The accumulation of lipids in circulating monocytes were detected with the NR lipid dye, which visualized both phospholipids and neutral lipids by high resolution confocal microscopy imaging (**Fig. 1A**). Neutral lipids (CEs and TGs) are shown in green, whereas phospholipids are shown in red. In order to distinguish between membrane-bound cholesterol (32) and neutral LDs, the fluorescent intensity of membrane-bound cholesterol was reduced until LDs alone were visible (supplemental Fig. S1A). The advantage of the phospholipid staining is that it results in a clear plasma membrane staining that can be used to assess whether neutral lipids are stored within cell bodies, whereas other lipid dyes often require alternative fluorescent labels for this purpose (33). To test whether NR staining was equivalent to ORO, we examined lipid accumulation in isolated monocytes (exposed to 50 μg/ml LDL for 1 h) and stained LDs using both ORO and NR. Imaging of ORO-stained monocytes using bright-field microscopy (Fig. 1B, left image; LDs indicated with white arrows) and confocal microscopy (Fig. 1B, right image) show intracellular LDs in red and green, respectively, whereas NR imaging of LDs of the same monocyte pool is shown in Fig. 1C. As can be seen, both NR and ORO were able to stain intracellular LDs. However, the use of methanol in the ORO staining procedure promotes fusion of LDs (34, 35).

It is crucial to determine whether LDs are located within the isolated monocytes because the presence of artifacts (i.e., background signal outside of the cell perimeter) may result in false positive LD quantification. For this reason, we either incubated isolated monocytes with or without freshly isolated LDL (50 μg/ml) and made an extensive z-stack that was subsequently modeled into a 3D structure in order to establish the LD localization in great detail (Fig. 1D; supplemental Movies S1, S2). Addition of LDL resulted in elevated intracellular neutral lipids when compared with nontreated monocytes, indicating that the lipid content of LDL particles is routed toward LDs. This concomitantly increased intracellular LD content.

Thus, NR staining allowed us to visualize elevated intracellular neutral LDs upon LDL exposure to monocytes. To investigate neutral LD composition, we isolated LDs from isolated circulating monocytes that were incubated with human LDL (0, 1, or 2 mg/ml apoB). Next, neutral lipids were separated from phospholipids and analyzed on HPLC. This revealed a dose-dependent increase in CE/TG ratio (CE/TG 3.46, 8.28 vs. 1.00-fold for 1, 2 vs. 0 mg/ml LDL, respectively; supplemental Fig. S1B). This implies that increased lipid loading of monocytes corresponds with elevated CE content upon monocyte exposure to LDL.

### Validation of NRQ

**Fig. 2.**
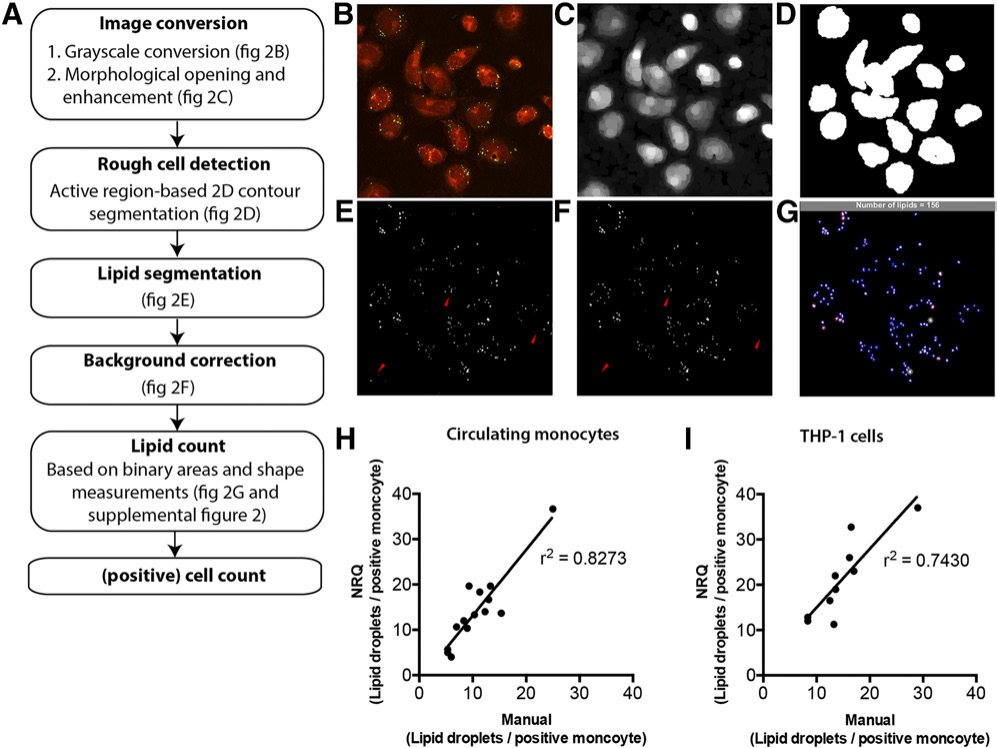
Processing steps and validation of the NRQ. A: Overview of processing steps of NRQ. B: Original image of the NR-stained monocytes. C: After converting it to grayscale, morphological opening of the grayscales and enhancement is created. D: Image (B) is then used to segment the cells with a region-based active contour method. E: Segmentation of the original image using a HSV color coding segmentation. F: After background correction using image (C), image (F) is obtained. Segmented lipid areas are then calculated with the aid of shape measurements (supplemental Fig. S3A–E). G: Counted areas are visualized with a blue circle, areas counted as two show an additional red circle, whereas areas counted as three are indicated with a green circle. The total amount of calculated LDs is shown in the gray bar; H, I: Pearson’s correlation between manual and NRQ quantification of LDs in circulating monocytes (H) and THP-1 cells (I) using 10–14 independent images.

Automated quantification of NR-stained monocytes provides a tool for detecting neutral LDs in a standardized and nonarbitrary manner. Here, we created and used a semi-automated segmentation to quantify LDs in the cells using the NRQ. The multiple NRQ processing steps are shown in a flow diagram (**Fig. 2A**). Obtained microscopic images (Fig. 2B) were converted into grayscale and subsequently contrast-enhanced (Fig. 2C). Next, LD count of cells in the perimeter of the image were excluded due to insufficient lipid count in these areas (Fig. 2D). After counting all LDs found in the FOV (Fig. 2E), based on the green channel (neutral lipids) in the RGB image, all LDs located outside the perimeter of the phospholipid monocyte membrane were excluded from the calculations, a process also known as background correction (Fig. 2F). Although LDs in isolated monocytes are spherically shaped in general, the space between LDs can be small and segmentation can lead to overlapping lipids. To distinguish individual LDs from connected LDs in the mask, shape measurements were computed. Next, thresholds were set for these measurements to complete LD count (supplemental Fig. S3A–E). LD count was visualized by individual LDs with a blue marked “x” and segmented areas that were counted twice and three times are displayed with a red circle and green circle, respectively (Fig. 2G). Monocytes were then counted by creating marks close to the center of each monocyte. Subsequently, the original image is displayed together with the transparent monocyte cell-mask. Next, lipid-positive monocytes were counted by subtracting the inverted binary lipid-mask of the new monocyte cell mask. Every region in the new monocyte cell mask that lost area pixels is now considered positive (yellow circle; supplemental Fig. S3A–E).

### Method validation

For a comprehensive validation of the methodology, we used the FDA Guideline for Industry (35). Here, the following sections will be discussed: *a*) precision, *b*) reproducibility, and *c*) accuracy.

### Repeatability and reproducibility

To assess repeatability of the method, as shown in supplemental Fig. S2, the obtained blood from one single donor (circled 1 in figure) was split into two equal portions (circled 2) for monocyte isolation (circled 3). These monocytes were divided over three microscope slides (circled 4) and were stained, imaged, and manually analyzed by the same observer during the same day (circled 5). The inter-experiment agreement was good for lipid-positive monocytes and LDs per positive monocyte, as indicated by ICC values of >0.86 (supplemental Table S1).

In order to address reproducibility, we isolated monocytes from one individual and divided these over six microscope slides. These six microscope slides were subsequently imaged on two occasions by two independent observers and counted manually. Agreement of the inter-observer was excellent for lipid-positive monocytes and LDs per positive monocyte, as indicated by ICC values of >0.91. The intra-observer agreement ICC displays good values of >0.87 (supplemental Table S1).

### Accuracy

In order to address the accuracy, we determined the correlation between NRQ and manually counted LDs. Monocytes were isolated as described previously and prepared for NR staining. Subsequently, at least six images per sample were prepared and quantified. This was done by both manual counting and NRQ-based counting. Subsequently, the LD counts per monocyte were plotted against each other in order to perform a linear regression analysis. As depicted in Fig. 2H, I, an r^2^ of 0.8273 was found for circulating monocytes, whereas THP-1 cells showed an r^2^ of 0.7430. Although, the r^2^ of both graphs indicated that the NRQ was accurate when compared with manual counting of LDs, the amounts of LDs counted by the NRQ tended to be higher compared with the manually determined LD count. This phenomenon most likely reflected the accuracy of detecting LDs by the NRQ. As mentioned in the Materials and Methods, LDs were segmented using a binary mask and subsequent HSV segmentation was used to set a specific color by which LDs were considered as positive LDs. The NRQ can more precisely determine whether a sample is positive. Therefore, manually counted LDs can be readily missed compared with the NRQ-counted LDs.

### Ex vivo lipid uptake by human peripheral CD14+ monocytes

**Fig. 3.**
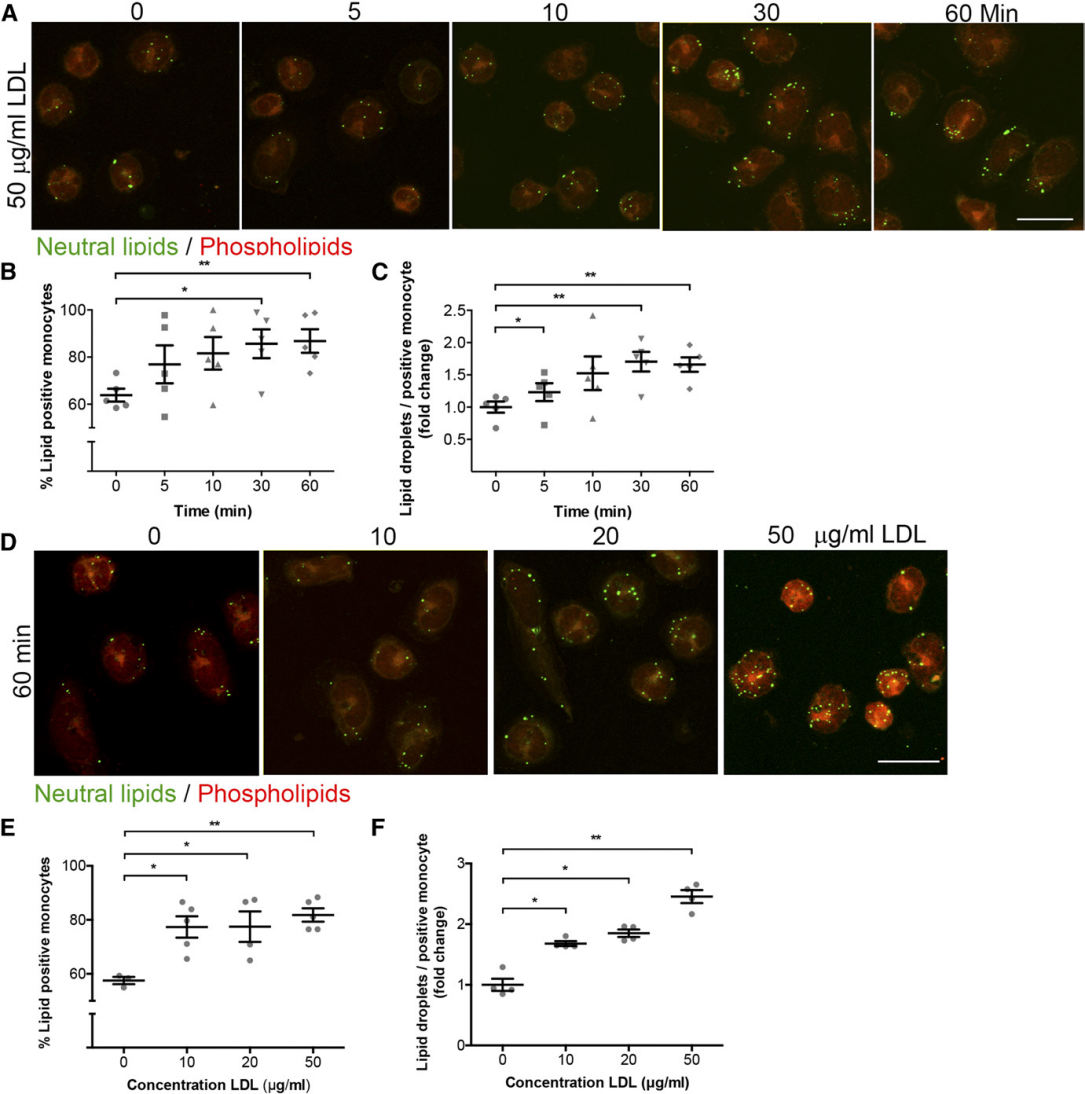
NR detection of LDs in monocytes in a time- and dose-dependent manner. A: Representative images of monocytes treated with 50 μg/ml LDL at different time points, as indicated. Scale bar, 10 μm. LDs and phospholipids are shown in green and red, respectively. B: Quantification of LD-positive monocytes after NR staining. C: Quantification of the amount of neutral LDs per positive monocyte after NR staining. D: Representative images for dose-dependent uptake of different concentrations of LDL, as indicated, for 1 h. Scale bar, 10 μm. LDs are shown in green, phospholipids in red. E, F: Quantification of dose dependency experiments in amount of LD-positive monocytes (E) and number of LDs (F) per LD-positive monocyte. *P* < 0.05 is considered significant. Data are shown as mean ± SEM for n = 5–7 independent experiments.

To assess whether NR, as quantified by NRQ, was able to detect differences in lipid uptake in monocytes, a time course experiment was conducted. Isolated monocytes were incubated with 50 μg/ml LDL for different times, as indicated, and lipid accumulation (**Fig. 3A**) was subsequently analyzed by assessing the increase in intracellular LDs and the percentage of lipid-positive cells (Fig. 3B, C, respectively). After 5 min of incubation with LDL, a significant increase in LDs per positive monocyte was already found (1.231 ± 0.139-fold; Fig. 3C). As shown in Fig. 3B, the number of lipid-positive monocytes after 30 min (85.6 ± 6.12%) and 60 min (86.8 ± 5.00%) significantly increased compared with baseline (63.8 ± 2.72%). This coincided with an increase in LDs per LD-positive monocyte (Fig. 3C) after 30 min (1.705 ± 0.151-fold) and 60 min (1.660 ± 0.112-fold). Altogether, the LDL uptake indicates, in a time-dependent manner, that NR detects rapidly formed LDs upon LDL exposure in circulating monocytes.

Next, we examined the LDL uptake in a dose-dependent manner (Fig. 3D). An increase in both lipid-positive monocytes and in LD content per LD-positive monocyte was observed after 60 min of incubation with either 10, 20, or 50 μg/ml freshly isolated LDL (Fig. 3E, F). These data suggest that LDL is taken up by monocytes leading to intracellular lipid body formation detectable by NR. To determine whether LDL uptake is mediated by receptors, the cells were incubated with 50 μg/ml LDL and either fixed with 4% paraformaldehyde or incubated at 4°C to induce metabolic arrest (supplemental Fig. S4A, B and supplemental Fig. S4C, D, respectively). As expected, both receptor-mediated uptake and passive diffusion were involved.

### Clinical validation of NRQ

**Fig. 4.**
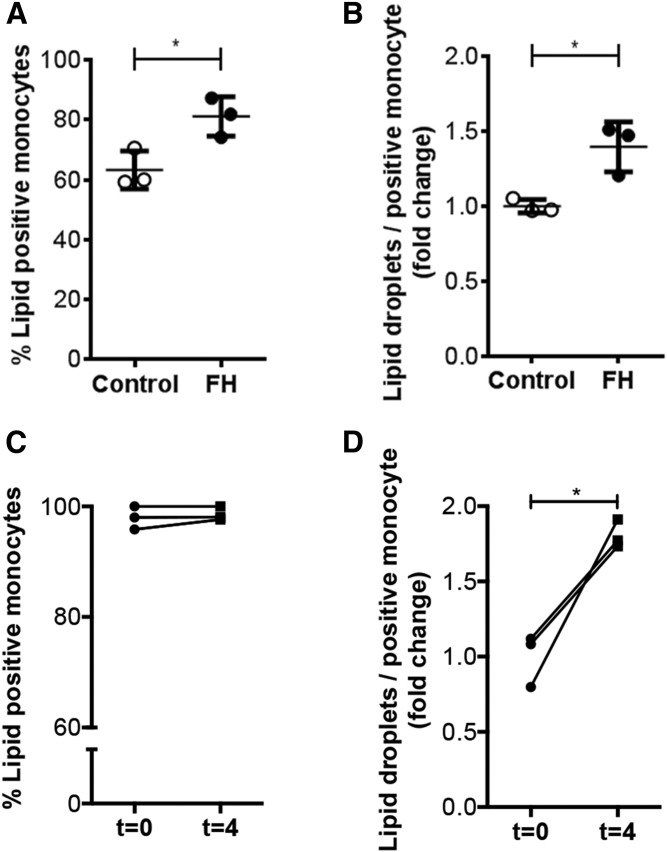
Clinical validation of NRQ. A, B: LD-positive monocytes (A) and LDs per LD-positive monocyte (B) in FH patients. C, D: LD-positive monocytes (C) and LDs per LD-positive monocyte (D) before and 4 h after oral fat administration. *P* < 0.05 considered significant. Data are shown as mean ± SD for n = 3 patients per group.

To examine the clinical applicability of NRQ, lipid accumulation in monocytes isolated from both FH patients and obese subjects who received an oral fat load were analyzed. Patients with FH had significantly elevated total and LDL cholesterol levels compared with age- and gender-matched healthy controls, but comparable TG levels. Obese subjects showed a mild increase in TG levels upon oral ingestion of fat (summarized in Table 1). Analyses of monocyte lipid content using the NRQ showed that FH patients had a significant increase in neutral LD-positive monocytes (63 ± 6.3% for healthy subjects vs. 81 ± 6.6% for FH patients; **Fig. 4A**) and neutral LDs per monocyte (1.395 ± 0.167-fold; Fig. 4B) increased in FH patients compared with healthy controls (1.000 ± 0.045-fold; Fig. 3B). Monocytes from obese subjects, isolated 4 h postprandially, showed a strong increase in neutral LD accumulation per LD-positive monocyte (1.806 ± 0.094-fold) compared with fasting state monocytes (Fig. 4D), although no differences were found in neutral LD-positive monocytes between groups (Fig. 4C), most likely caused by increased baseline TG levels in obese subjects. This clinical pilot indicates that NRQ is able to detect in vivo differences in neutral LD content in circulating monocytes.

## DISCUSSION

Here, we showed that the lipid dye, NR, is suitable for assessment of intracellular lipid content in isolated circulating monocytes and we subsequently developed an automated LD quantification tool.

### Benefits of NR

In the present study, NR staining was successfully used for detecting alterations in LDL-induced neutral lipid accumulation over time (Fig. 3A–C) and in a concentration-dependent manner (supplemental Fig. S1B; Fig. 3D–F). The validation of NR-based LD measurement revealed that it is a reliable tool to detect alterations in LD accumulation in circulating monocytes. NR has several advantages over other lipid probes or dyes: *a*) The lipophilic dye, NR, is inexpensive as compared with antibody-based lipid probes. *b*) Cellular NR staining is quick and easy to apply, whereas antibody-based procedures are time consuming due to the multiple staining steps. *c*) The fluorescence maxima fluctuate based on the hydrophobicity of NR allowing discrimination between neutral lipids and phospholipids. This enables specific detection of intracellular neutral lipids stored in LDs, whereas a lipid dye, such as BODIPY, may bind nonspecifically (16). Interestingly, NR stains phospholipids, which allows visualization of the cell membrane (22). *d*) NR is an uncharged heterocyclic molecule and, thus, soluble in organic solvents (21). Furthermore, the use of confocal microscopy allows us to visualize LD at different focal planes within monocytes. This is in contrast to ORO, a frequently used lipid dye that is imaged using a bright-field microscope, which often cannot image in the Z-dimension. Without this dimension, it is more difficult to count and quantify all the lipids within one cell, because most LDs are not located in one focal plane, resulting in potential difficulties in the quantification of LD content. In addition, the ORO-staining procedure causes LDs to fuse with each other causing unreliable LD assessment (17). *e*) Further validation of NR applicability revealed its usefulness in staining of other cell types, such as THP-1 cells, murine bone marrow-derived macrophages (data not shown), and human fibroblasts (data not shown).

### Automated image analysis using NRQ

Because of its sensitivity, the NRQ quantification tool allows the analysis of time-dependent increase of LD content. We showed that NRQ is suitable to replace manual counting of intracellular LDs in circulating monocytes, as well as THP-1 cells (Fig. 2H, I, respectively) when high quality images were captured, because low resolution of images complicated detection of green fluorescent LDs (data not shown). In addition, both NRQ and the confocal settings used should remain identical within a study to perform objective analysis. For optimal use of NRQ, one must take caution concerning the amount of cells per FOV. Too many cells per FOV could result in cell clusters that are too dense, leading to difficulties in semi-automated quantification of single cells (data not shown). Therefore, we recommend maximal seeding of 1 × 10^5^ monocytes/cm^2^ for optimal use of NRQ. Furthermore, the r^2^ of both graphs (Fig. 2H, I) indicated that the NRQ is accurate when compared with manual counting of LDs; the amounts of LDs counted by the NRQ tend to be higher compared with the manually determined LD count. This phenomenon was most likely caused by the accuracy of detecting LDs by the NRQ. As mentioned in the Materials and Methods, LDs were segmented using a binary mask and subsequent HSV segmentation was used to set a specific color by which LDs were considered as positive LDs. The NRQ can therefore strictly determine whether a sample is positive or not, where manual detection of LDs remains arbitrary. In addition, manually counted LDs are more easily missed compared with the NRQ-counted LDs. Therefore, NRQ-counted LDs tend to be slightly increased when compared with LDs counted manually.

### Implications for clinical research

Research into lipid accumulation focused predominantly on plaque macrophages; however, recent findings highlight the importance of the interaction between lipoproteins and mononuclear cells already in the circulation (28, 29, 37). Foamy monocytes have been shown to contribute to nascent atherosclerosis (38). Intracellular lipid accumulation induces β2-integrin (CD11c/CD18) surface expression (39), which plays an important role as a compatible structure for vascular cellular adhesion molecules on endothelial cells, supporting monocyte recruitment into atherosclerotic plaques (6, 40). Although it is not in the scope of this work, it would be interesting to investigate how monocytes are able to internalize LDL particles in a short period of time and when monocytes become saturated upon lipid loading, as can be seen in Fig. 3B, C, E, and F. LD accumulation of monocytes tended to show rapid uptake (after 5 min) and saturation after 10 min of LDL incubation. This might be attributed to the lack of efflux mechanisms in an ex vivo experimental setup. This absence of plasma cholesterol efflux mechanisms, such as high density cholesterol, may induce an accelerated accumulation response. This is due to the lack of opportunities to efflux cholesterol (41). To substantiate this, it was found that the absolute amount of LDs starting at 10 min and higher of ex vivo LDL incubation did not differ from the amount of LDs found in monocytes derived from FH patients. This implies that the lack of cholesterol efflux mechanisms leads to quick LD saturation in monocytes (data not shown). Foamy monocytes or lipid-laden monocytes are currently intensively studied, making NRQ a valuable tool for future research. The clinical potential of NRQ was further substantiated via pilot experiments showing that it was indeed capable of detecting increased monocyte neutral LD content in FH patients. Also, in obese subjects who underwent an oral lipid challenge, an increase in neutral LDs in circulating monocytes was demonstrated (Fig. 4).

### Limitations

Although NR staining and NRQ analysis offer a reproducible method for intracellular quantification of LD content, there are some limitations to discuss. One possible limitation is the broad emission spectrum of the dye, because fluorescent signal is observed from 488 nm up to the far red spectrum (42). It is possible however, to use the dye in combination with fluorophores, which can be excited in the violet spectrum (405 nm), in order to perform dual localization studies using NR (43, 44). The diameter of LDs in nonadipocytes generally range from 0.1 μm, but can be up to 100 μm in size in white adipocytes (45). Because NRQ was developed to detect LDs based on fluorescent intensity and their correlated pixel size, NRQ was solely validated to image LDs in the nonadipocyte range when recorded with 1,024 × 1,024 pixels. Based on this study, we recommend imaging cells with a 63× objective and a 1.5–2× digital zoom for optimal usage of the NRQ.

## CONCLUSIONS

Altogether, we provide a new quantitative tool, the NRQ, for quick and reproducible NR-based lipid detection in isolated patient-derived circulating monocytes. NRQ measures the total number of lipids per FOV and the number of LD-positive cells. With these parameters, one can quantify LD content on a per cell basis and the percentage of lipid positive cells. We also revealed that, besides circulating monocytes, NRQ is also applicable for THP-1 cells, suggesting its potential for other cell types. Additionally, the NR staining procedure is sensitive enough to detect any changes in LD dynamics and NRQ is a useful tool for analysis of shifts in neutral LD content in patient-derived circulating monocytes. In addition, NRQ could be helpful to standardize the field of LD imaging and analysis. Furthermore, it would be interesting to see whether increased LD accumulation and, hence, increased pro-inflammatory phenotype could be used as a possible biomarker for cardiovascular risk assessment.

## Supplementary Material

Supplemental Data
