# Supplementary material for: Nile Red Quantifier: a novel and quantitative tool to study lipid accumulation in patient-derived circulating monocytes using confocal microscopy
Source: J Lipid Res. 2017 Sep 28;58(11):2210–9. doi: 10.1194/jlr.D073197 (PMC5665660; doi:10.1194/jlr.D073197)
Supplement: Supplemental Data [file supp_58_11_2210__index.html]

Nile Red Quantifier: a novel and quantitative tool to study lipid accumulation in patient-derived circulating monocytes using confocal microscopy — Nile Red Quantifier: a novel and quantitative tool to study lipid accumulation in patient-derived circulating monocytes using confocal microscopy — Supplemental Data 

# Nile Red Quantifier: a novel and quantitative tool to study lipid accumulation in patient-derived circulating monocytes using confocal microscopy

## Supplemental Data

- Movie 1: High lipid uptake (.mov, 8.9 MB) - LDL-lipid uptake increased intracellular LDL-mediated LD formation in circulating monocytes. Monocytes were stimulated with 50 g/ml LDL and 3D-modeling revealed an elevated lipid uptake
- Movie 2: Low lipid uptake (.mov, 4.6 MB) - Supplemental Movies LDL-lipid uptake increased intracellular LDL-mediated LD formation in circulating monocytes. Monocytes were unstimulated
- Supplemental data (.pdf, 2.2 MB) - Supplemental data
