# Supplementary material for: Nile Red Quantifier: a novel and quantitative tool to study lipid accumulation in patient-derived circulating monocytes using confocal microscopy
Source: J Lipid Res. 2017 Sep 28;58(11):2210–9. doi: 10.1194/jlr.D073197 (PMC5665660; doi:10.1194/jlr.D073197)
Supplement: Supplemental Data [file 10.1194_D073197_jlr.D073197-3.pdf]

Supplemental data

Supplementary methods NRQ

Shape measurements performed on the lipid mask are; area, number of pixels in every region; perimeter, scalar that specifies distance around the boundary of the pixel region, solidity, scalar that is area divided by convex area (number of pixels that specifies the smallest convex polygon containing the region); bounding box, axis sizes of smallest rectangle containing area; eccentricity, scalar that specifies the eccentricity of the ellipse (Supplemental Figure S3). Some of the mentioned measurements are calculated using other measurements that follows out of all the shape measurements resulting from regionprops.m, a standard function from MATLAB (The MathWorks Inc., Natick, MA, United States). For example, the major axis length (Supplemental Figure S3B) of the mask is measured from which the eccentricity (a scalar) can be calculated using the ratio of the foci of the ellipse and the major axis length. The value can be between 0 and 1, in which an ellipse with an eccentricity of 0 actually is a circle and an eccentricity of 1 would mean a line segment. For individual lipids, the expectation for eccentricity is close to 0 and if it is close to 1 it is more likely to be a connected mask as lipids are thought to be more round shaped due to their hydrophobic core (1).

Upon inspection of individual lipids in the segmented lipid mask, it is clearly noticed that some lipid areas in the mask were connected and did not represent one lipid droplet. Therefore, because the calculation of lipids is based on individual areas in the mask, shape measurements could provide that extra information on discriminating lipids from one another. In Supplemental Figure S2A one can see an enlargement of two segmented areas in the lipid mask, which by human eye could be explained as three lipids. Measurements as major axis length, for calculating eccentricity (2), is performed as can be seen in Figure S2. The convex image is a rectangular cut-out version of the smallest polygon sized pixel region, adding more information about size and form of the segmented area (Supplemental Figure S2C). With these measurements, we can discriminate the lipids that are spherically shaped (thus one lipid) from those who are not or extremely large and thus are most likely to represent more than one lipids.

These different shape properties are used to set conditions for the analysis of the segmented regions. When these shape measurements are above all set thresholds, determined by visual inspection and exploration of the data, these lipids will be counted twice and marked (extra) with a red circle. In

rare cases, if the size of the lipid mask exceeds 40 pixels (‘AreaThreshold2’), 5 times the size of the average lipid, it will be counted as three lipids (maximum count of one mask) and additionally marked by a green circle (Supplemental Figure S2D-E). This method allows the user to more accurately count individual lipids.

Below is a list of the set thresholds for each measured parameter:

AreaThreshold1 = 16 (pixels)

AreaThreshold2 = 40 (pixels)

PerimeterThreshold = 11 (means 11 or higher)

SolidityThreshold = 1 (means below 1)

CVXimageThreshold = 20 (means 20 or higher)

EccentricityThreshold = 0.6 (or higher but 0.6 was seen as high [2

References

1. Penno, A., G. Hackenbroich, and C. Thiele. 2013. Phospholipids and lipid droplets. *Biochim. Biophys. Acta.* **1831**: 589–594.

2. Weisstein, Eric W. "Eccentricity." From *MathWorld*--A Wolfram Web Resource. http://mathworld.wolfram.com/Eccentricity.html

Supplemental Figure S1

A

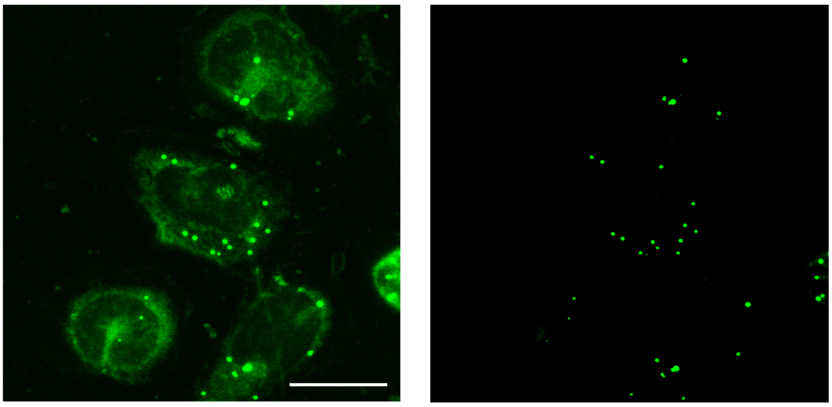

B

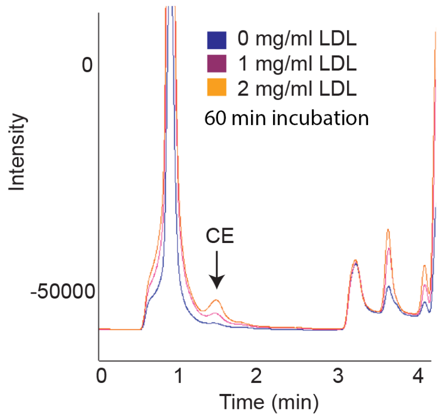

Normalized CE/TG ratio:
0 mg/ml LDL: 1,00 x fold increase
1 mg/ml LDL: 3,46 x fold increase
2 mg/ml LDL: 8,28 x fold increase

Supplemental Figure S1 A Green fluorescence reveals both membrane bound- and intracellular neutral lipids (left image; neutral lipids in green). For analysis of intracellular LDs, fluorescent intensity was decreased until solely LDs were visible (right image); **B** HPLC-analysis reveals elevated cholesterol ester content of monocytes upon LDL loading for 1h at 37°C and 5% CO_2. The relative peak areas of CE/TG ratio were calculated in monocytes stimulated with 0 (blue line), 1 (purple line) and 2 mg/ml LDL (orange line). CE/TG ratio increased in a dose-dependent fashion once stimulated with LDL. HPLC, high performance liquid chromatography; LDL, low-density lipoprotein; CE, cholesterol ester; TG, triglyceride; LD, lipid droplet.

Supplemental Figure S2

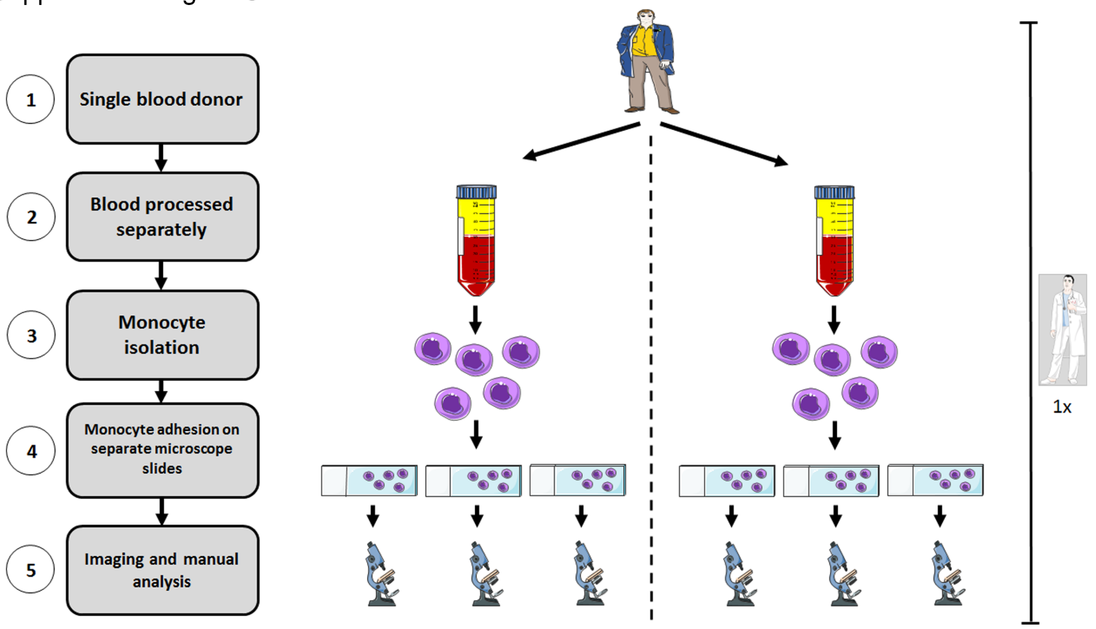

Supplemental Figure S2 Outline of inter-experiment methodology; from monocyte isolation to imaging Nile Red stained monocytes. Blood is obtained from a single healthy donor (1) and split into two portions (2 for monocyte isolation (3). These monocytes were divided over three microscope slides (4) and were stained, imaged and manually analyzed by the same observer during the same day (5). LD, lipid droplets; NR, Nile red; FN, fibronectin.

Supplemental Figure S3

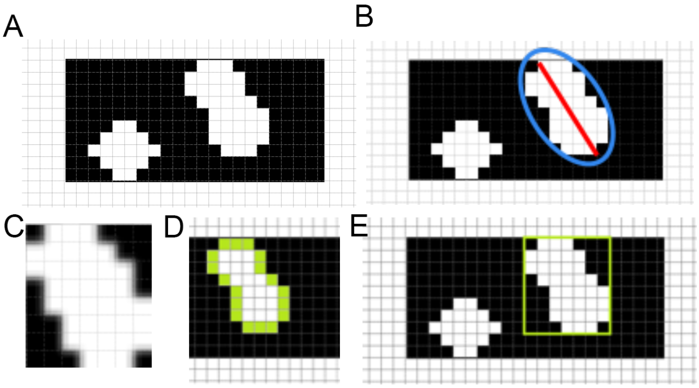

Supplemental Figure S3 Example of shape measurements applied on **A** Segmented lipid areas; **B** From the right area the major axis length is depicted by the red line across the area from which the eccentricity of the ellipse (blue) is calculated; **C** A small image which only contains the smallest polygon sized area of that segmented lipid area; **D-E** The perimeter and bounding box in green, respectively.

Supplemental Figure S4

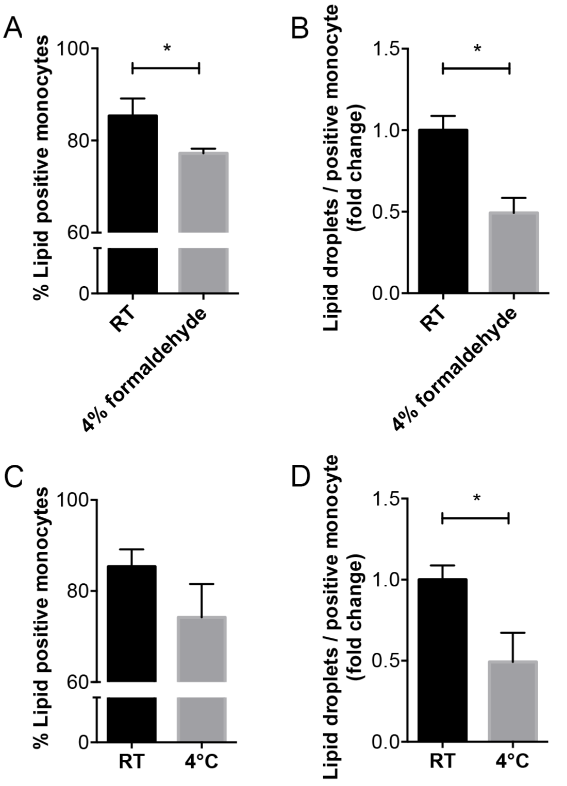

Supplemental Figure S4 Metabolic arrest leads to inhibition of intracellular LDL-initiated LD formation. **A-B** 4% Formaldehyde fixed samples showed less LD-positive monocytes (A) and less LDs per LD-positive monocyte compared to non-fixed monocytes; **C-D** Samples incubated at 4°C lead to decreased number of LD-positive monocytes (C) and declined number of LDs per LD-positive monocyte. LDL, low-density lipoprotein; LD, lipid droplet

Supplemental Table S1. Inter-experiment, intra- and inter-observer agreement

	% lipid positive monocytes	Lipids per monocyte
**Inter-experiment**		
Paired diff between exp 1 and exp 2	1,058 ± 0,039	1,123 ± 0,061
COV	3,1%	5,5%
Inter ICC (CI)	0,861 (0,406-0,968)	0,896 (0,266-0,079)
**Intra-observer**		
Paired diff between observation 1 and 2	1,011 ± 0,015	1,021 ± 0,099
COV	0,2%	4,7%
Intra ICC (CI)	0,995 (0,982-0,999)	0,877 (0,483-0,970)
**Inter-observer**		
Paired diff between observer 1 and 2	1,021 ± 0,067	0,9133 ± 0,091
COV	2,5%	1,7%
Inter ICC (CI)	0,961 (0,909-0,984)	0,919(0,808-0.966)

CI, confidence interval; COV, coefficient of variation; diff, difference; ICC, intraclass correlation coefficient

Supplemental Movies LDL-lipid uptake increased intracellular LDL-mediated LD formation in circulating monocytes. Monocytes were stimulated with 50 µg/ml LDL and 3D-modeling revealed an elevated lipid uptake (**1**) compared to unstimulated monocytes (**2**). Monocytes are shown in grey, whereas LD are in green. Scale bar, 7 µm.

LDL, low-density lipoprotein; LD, lipid droplet
